# Supplementary material for: Centrosome migration and apical membrane formation during epithelial polarization in MDCK cysts
Source: eLife. 2026 Sep 8;13:RP101088. doi: 10.7554/eLife.101088 (PMC13553070; doi:10.7554/eLife.101088)
Supplement: Supplementary file 2 [file elife-101088-fig3-figsupp1-data1.zip › Figure 3-figure supplement 1 - Source data 1/Figure 3-figure supplement 1F.pdf]

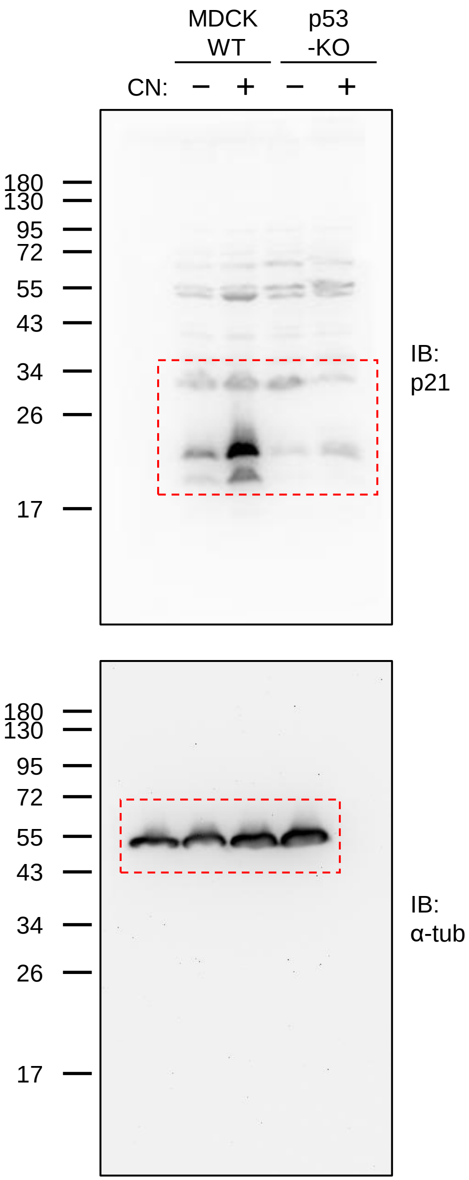

MDCK
WT
p53
-KO
CN: − + − +
180
130
95
72
55
43
34
26
17
IB:
p21
180
130
95
72
55
43
34
26
17
IB:
α-tub
