# Supplementary material for: Centrosome migration and apical membrane formation during epithelial polarization in MDCK cysts
Source: eLife. 2026 Sep 8;13:RP101088. doi: 10.7554/eLife.101088 (PMC13553070; doi:10.7554/eLife.101088)
Supplement: Supplementary file 4 [file elife-101088-fig4-figsupp2-data1.zip › Figure 4-figure supplement 2 - Source data 1/Figure 4-figure supplement 2G.pdf]

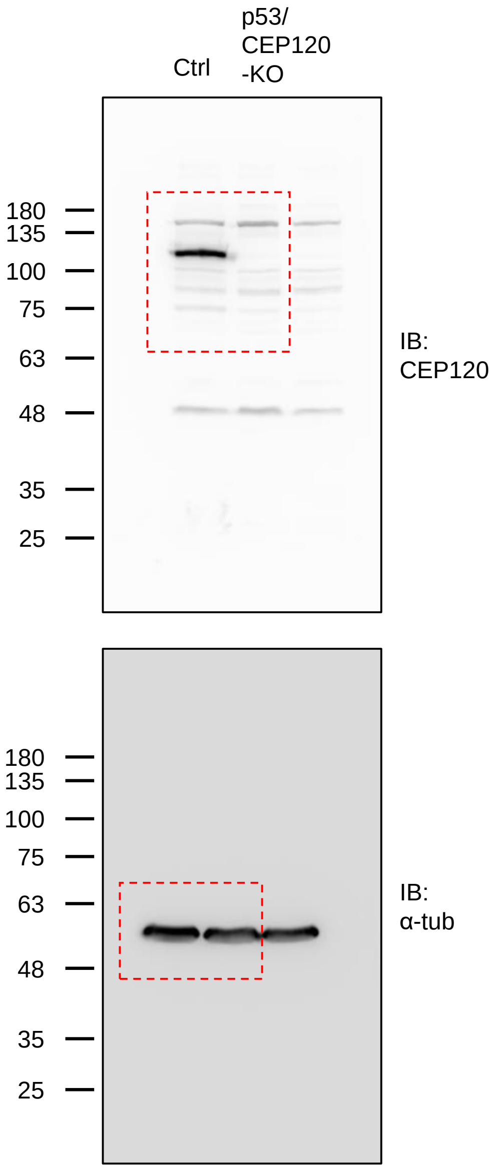
Ctrl
p53/
CEP120
-KO
180
135
100
75
63
48
35
25
IB:
CEP120
180
135
100
75
63
48
35
25
IB:
α-tub
