# Supplementary material for: Centrosome migration and apical membrane formation during epithelial polarization in MDCK cysts
Source: eLife. 2026 Sep 8;13:RP101088. doi: 10.7554/eLife.101088 (PMC13553070; doi:10.7554/eLife.101088)
Supplement: Supplementary file 6 [file elife-101088-fig5-figsupp1-data1.zip › Figure 5-figure supplement 1 - Source data 1/Figure 5-figure supplement 1.pdf]

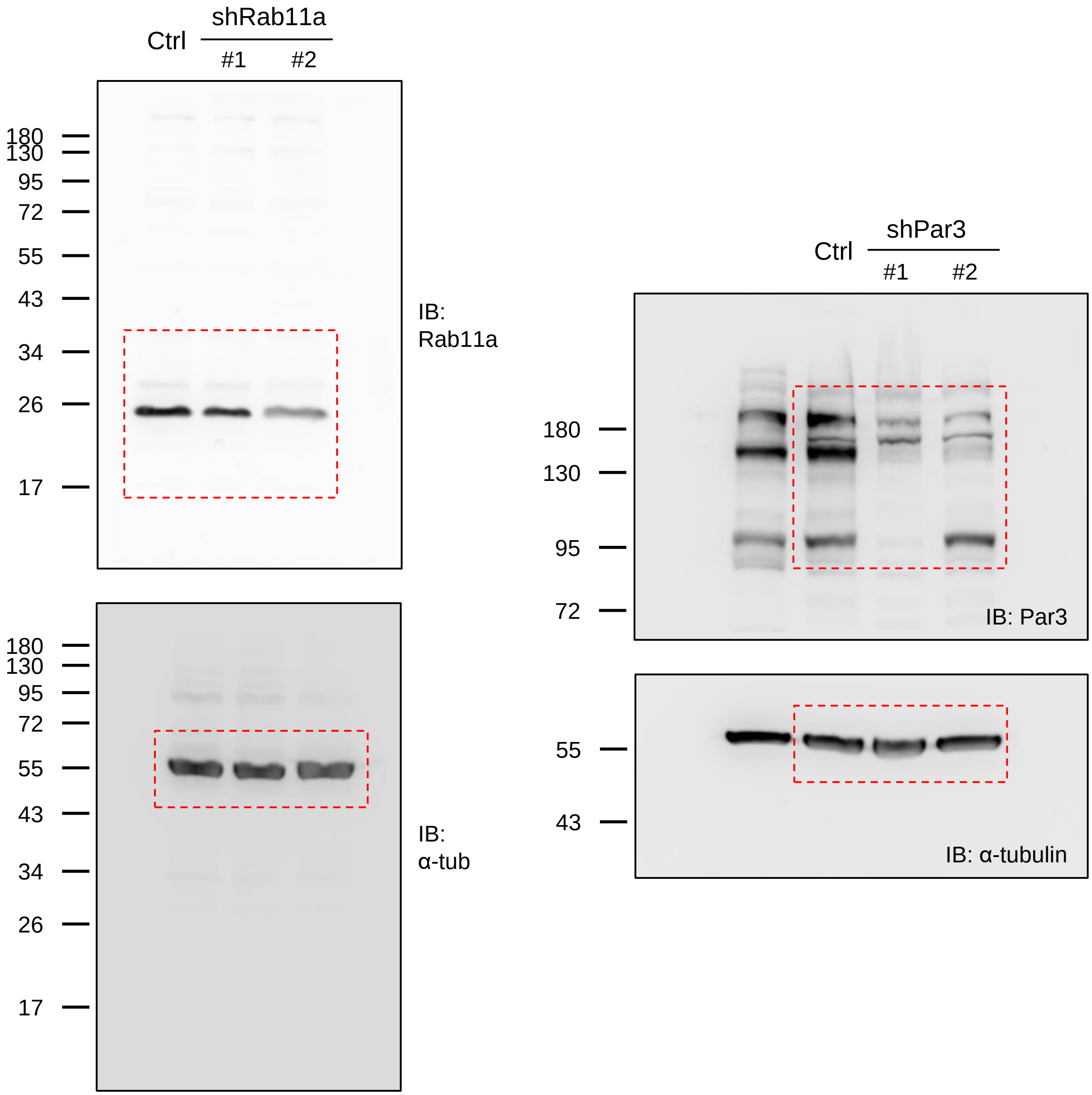
Ctrl
shRab11a
#1
#2
180
130
95
72
55
43
34
26
17
IB: Rab11a
180
130
95
72
55
43
34
26
17
IB: α-tub
Ctrl
shPar3
#1
#2
180
130
95
72
IB: Par3
55
43
IB: α-tubulin

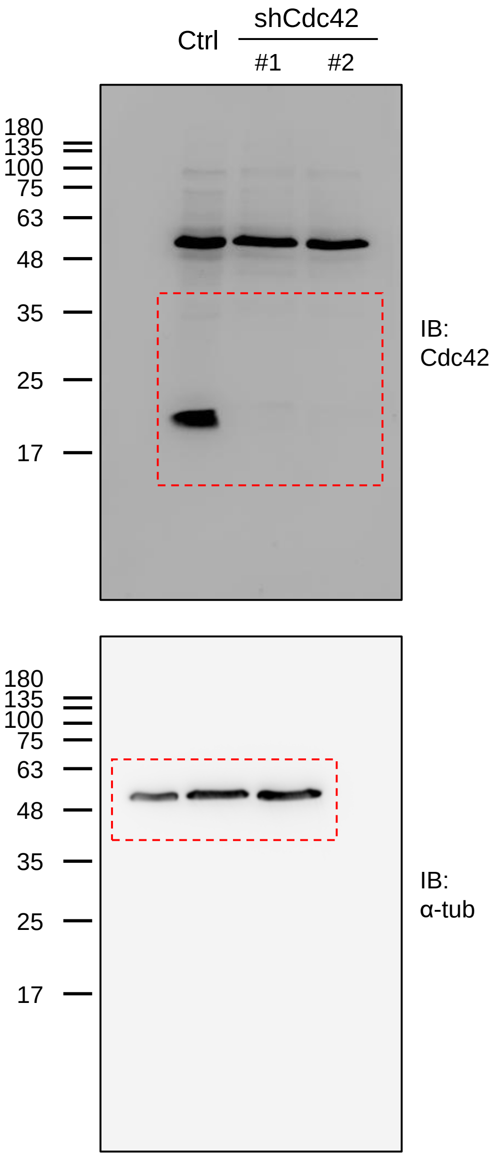
Ctrl
shCdc42
#1
#2
180
135
100
75
63
48
35
25
17
IB:
Cdc42
180
135
100
75
63
48
35
25
17
IB:
α-tub
