# Supplementary material for: Centrosome migration and apical membrane formation during epithelial polarization in MDCK cysts
Source: eLife. 2026 Sep 8;13:RP101088. doi: 10.7554/eLife.101088 (PMC13553070; doi:10.7554/eLife.101088)
Supplement: Supplementary file 8 [file elife-101088-fig5-figsupp2-data1.zip › Figure 5-figure supplement 2 - Source data 1/Figure 5-figure supplement 2E.pdf]

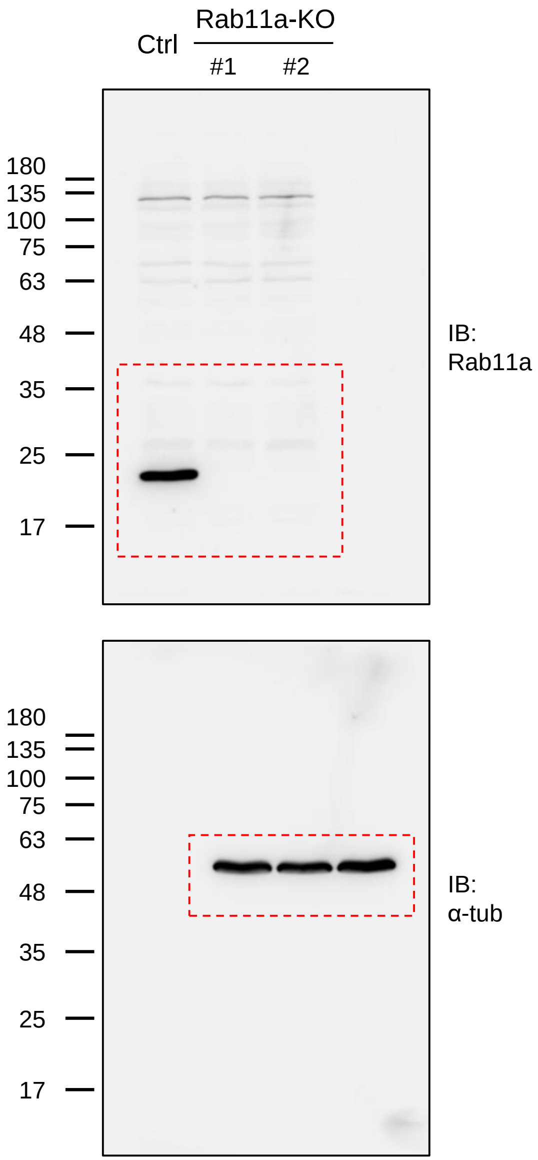
Rab11a-KO
Ctrl
#1
#2
180
135
100
75
63
48
35
25
17
IB:
Rab11a
180
135
100
75
63
48
35
25
17
IB:
α-tub
