# Supplementary material for: Centrosome migration and apical membrane formation during epithelial polarization in MDCK cysts
Source: eLife. 2026 Sep 8;13:RP101088. doi: 10.7554/eLife.101088 (PMC13553070; doi:10.7554/eLife.101088)
Supplement: Peer review history

# Editor report: eLife Assessment

**DOI:** 10.7554/eLife.101088.4.sa0

**Authors:** Heidi Hehnly

**Affiliations:** Syracuse University United States

**Keywords:** Convincing, Important

This **important** study contributes to our understanding of how epithelial cells establish polarity by identifying a hierarchy in which Par3 acts upstream of centrosome positioning and apical membrane initiation. The evidence supporting the main conclusions is **convincing**, and the authors addressed the unresolved questions about microtubule organization and the need for clearer integration of quantitative and conceptual points raised in review. The work will be of interest to cell and developmental biologists.

# Referee report: Reviewer #1 (Public review)

**DOI:** 10.7554/eLife.101088.4.sa1

Summary:

Wang, Po-Kai et al., utilized the de novo polarization of MDCK cells cultured in Matrigel to assess the interdependence between polarity protein localization, centrosome positioning and apical membrane formation. They show that the inhibition of Plk4 with Centrinone does not prevent apical membrane formation, but does result in its delay, a phenotype the authors attribute to the loss of centrosomes due to the inhibition of centriole duplication. However, the targeted mutagenesis of specific centrosome proteins implicated in the positioning of centrosomes in other cell types (CEP164, ODF2, PCNT and CEP120), as well as the use of dominant negative constructs to inhibit centrosomal microtubule nucleation did not affect centrosome positioning in 3D cultured MDCK cells. A screen of proteins previously implicated in MDCK polarization revealed that the polarity protein Par-3 was upstream of centrosome positioning, similar to other cell types.

Strengths:

The investigation into the temporal requirement and interdependence of previously proposed regulators of cell polarization and lumen formation is valuable. The authors have provided a detailed analysis of many of these components at defined stages of polarity establishment and well demonstrate that centrosomes are not necessary for apical polarity formation, but are involved in the efficient establishment of the apical membrane.

Weaknesses:

Key questions remain regarding the structure of the intracellular cytoskeleton following depletion of centrosomes, centrosome proteins, or abrogation of centrosome microtubule nucleation. The authors strengthen their model that centrosomes are positioned independently of microtubule nucleation using dominant negative Cdk5RAP2 and NEDD-1 constructs, however, the structure of the intracellular microtubule network remains unresolved and will be an important avenue for future investigation.

# Referee report: Reviewer #3 (Public review)

**DOI:** 10.7554/eLife.101088.4.sa2

Here the Wang et al resubmit their manuscript describing the events in the establishment of polarity in MDCK cells cultured in vitro. As with the original version, the description is throughout and is important to the field to report as it establishes a hierarchy of events in polarization, placing Par3 upstream of centrosome positioning and apical membrane component trafficking. Unfortunately, in the revised version, the authors addressed almost none of my points. They did a cursory job of responding in the rebuttal letter but made little attempt to actually address what was being asked or to incorporate any of my suggestions into the manuscript. The particularly egregious examples are cited below:

Comments on revisions:

(1) My original main experimental concern was not addressed: I had originally asked what role microtubules play in the process of polarization (either centrosomal or non-centrosomal). An obvious model is that Gp135, Rab11, etc. are delivered to the AMIS on centrosomal microtubules. Centrosomes might also be pulled to the AMIS via cortically derived microtubules as is the case in the *C. elegans* intestine where the centrosome moves apically on apical microtubules via dynein directed transport to the cortically anchored minus ends. The authors do not explore the role of microtubules in the revision, citing that it was not possible to observe the microtubules directly or to perform nocodazole experiments during polarization. Instead, the authors use a relatively new genetic tool to disrupt centrosomal microtubules. They appear to succeed in displacing centrosomal g-tubulin using this tool, but without being able to observe microtubules, a remaining caveat of this experiment is that it is still unclear whether the authors have removed centrosomal microtubules. Compounding this issue is that this tool has never been used in MDCK cells. The authors conclude "we found that cells lacking centrosomal microtubules were still able to polarize and position the centrioles apically.", but they have not shown this, instead the data suggest this conclusion and the authors should acknowledge the caveat that they have no idea whether centrosomal microtubules are abolished. Similarly, the authors also state: "Additionally, although PCNT knockout cells show reduced microtubule nucleation ability, they still recruit a small amount of γ-tubulin". Where are the data that show that microtubule nucleation is reduced in these PCNT knock out cells?

(2) Many of my comments were addressed in the rebuttal, but not in the text.

The non-centrosomal GP135 in Figure 2 is not acknowledged or explained.

That the polarity index does not actually measure polarity, but nuclear-centrosome distance is not acknowledged or explained in the paper.

I still don't believe that the quantification in Figure 3D matches the images I am being shown in Figure 3A. In the centrinone treatment condition, there is certainly an enrichment of GP135 at the AMIS that is not detected in the quantification. The method described in the rebuttal might miss this enrichment if it is offset from line drawn between the centroid of the two nuclei.

Cell height changes in the centrosome depleted cysts are still referenced in the text ("the cell heights of the centrosome-depleted cysts are less uniform"), but no specific data or image is called out. Currently, Figure 3G is referenced, but that is a graph of GP135 intensity.

In my original review, I called on the authors to comment on the striking similarity of the mechanisms they documented in MDCK cells to what has been shown in in vivo systems. The authors did not do this, instead restating in the rebuttal some features of what they found. But the mechanisms shown here are remarkably similar to the polarization of primordia that generate tubular organs in vivo. Perhaps most striking is the similarity to the C> elegans intestine where Par3 localizes to the cortex at the site of an apical MTOC that pulls the centrosome to the apical surface via dynein (Feldman and Priess, 2012). Instead of discussing this similarity, the authors state: "Par3 is likely to regulate centrosome positioning through some intermediate molecules or mechanisms, but its specific mechanism is still unclear and requires further investigation." Given the acetylated tubulin signal emanating from the Par3 positive patch in Figure 5E and F, I suspect similar mechanisms to the *C. elegans* intestine are at play here. Such a parallel should be noted in the Discussion.

I had originally commented that "I find the results in Figure 6G puzzling. Why is ECM signaling required for Gp135 recruitment to the centrosome. Could the authors discuss what this means?" The authors responded that "The data in Figure 6G do not indicate that ECM signaling is required for the recruitment of Gp135 to the centrosome". In Figure 6G, the localization of GP135 to the centrosome appears significantly delayed compared to its localization to the centrosome in images where cells were cultured in Matrigel. Indeed, the authors argue that the centrosomal localization precedes and contributes to its localization to the AMIS. In the absence of ECM, GP135 localizes to the membrane before it localizes to the centrosome and its localization to the centrosome appears significantly reduced. Thus, my original and current interpretation is that ECM signaling is somehow required for the centrosomal targeting of GP135. One could make a competition argument, i.e. that the cortex in the absence of ECM is somehow a more desirable place to localize than the centrosome, but this experiment also argues that the centrosome does not need to be a source of this material in order for it to end up on the cortex.

(3) There needs to be precision in the language used in many places:

I don't understand this line in the abstract: "When cultured in Matrigel, de novo polarization of a single epithelial cell is often coupled with mitosis." If a cell has divided, it is no longer a single cell.

The authors state in the Introduction "Because of its strong ability to nucleate microtubules, the centrosome functions as the primary microtubule organizing center", but then state ""In polarized epithelial cells, the centrosome is localized at the apical region during interphase, which contributes to the construction of an asymmetric microtubule network conducive to polarized vesicle trafficking". In the latter statement, I assume the authors are describing the well-characterized apical microtubule network in epithelial cells that is non-centrosomal. Thus, the latter sentence is at odds with the former.

The authors continually refer to Par3 as a tight junction protein. "Par3, which controls tight junction assembly to partition the apical surface from the basolateral surface". To my knowledge, PARD3 is an apical protein with similar localization to *C. elegans* PAR-3 and Drosophila Bazooka. PARD3B is a junctional protein. I assume that the antibody that the authors are using is to PARD3 and not PARD3B? Can the authors please clarify this in the text.

# Author comment: Author response

**DOI:** 10.7554/eLife.101088.4.sa3

**Authors:** Po-Kai Wang, Keng-Hui Lin, Tang K Tang

**Affiliations:** Institute of Biomedical Sciences, Academia Sinica Taipei Taiwan; Institute of Physics, Academia Sinica Taipei Taiwan; Institute of Biomedical Sciences, Academia Sinica Taipei Taiwan

The following is the authors’ response to the previous reviews

**Public Reviews:**

**Reviewer #1 (Public review):**

Summary:

Wang, Po-Kai et al., utilized the de novo polarization of MDCK cells cultured in Matrigel to assess the interdependence between polarity protein localization, centrosome positioning and apical membrane formation. They show that the inhibition of Plk4 with Centrinone does not prevent apical membrane formation, but does result in its delay, a phenotype the authors attribute to the loss of centrosomes due to the inhibition of centriole duplication. However, the targeted mutagenesis of specific centrosome proteins implicated in the positioning of centrosomes in other cell types (CEP164, ODF2, PCNT and CEP120), as well as the use of dominant negative constructs to inhibit centrosomal microtubule nucleation did not affect centrosome positioning in 3D cultured MDCK cells. A screen of proteins previously implicated in MDCK polarization revealed that the polarity protein Par-3 was upstream of centrosome positioning, similar to other cell types.

Strengths:

The investigation into the temporal requirement and interdependence of previously proposed regulators of cell polarization and lumen formation is valuable. The authors have provided a detailed analysis of many of these components at defined stages of polarity establishment, and well demonstrate that centrosomes are not necessary for apical polarity formation, but are involved in the efficient establishment of the apical membrane.

Weaknesses:

Key questions remain regarding the structure of the intracellular cytoskeleton following depletion of centrosomes, centrosome proteins, or abrogation of centrosome microtubule nucleation. The authors strengthen their model that centrosomes are positioned independently of microtubule nucleation using dominant negative Cdk5RAP2 and NEDD-1 constructs, however, the structure of the intracellular microtubule network remains unresolved and will be an important avenue for future investigation.

We thank the reviewer for raising this important point. We agree that understanding the organization of the intracellular microtubule network following centrosome depletion, disruption of centrosomal proteins, or inhibition of centrosomal microtubule nucleation will be important for further mechanistic insight. However, a detailed analysis of cytoskeletal architecture under 3D culture conditions would require super-resolution or other advanced microscopy techniques and therefore falls beyond the scope of the current study. Nevertheless, several previous studies conducted under conventional 2D culture conditions provide relevant mechanistic context for interpreting our findings.

(1) Centrosome depletion by centrinone treatment

Previous studies have shown that upon centrosome loss induced by the Plk4 inhibitor centrinone, alternative microtubule-organizing centers (MTOCs), particularly the Golgi apparatus, can compensate by nucleating non-centrosomal microtubules (Chen et al., 2022; Gavilan et al., 2018; Martin, Veloso, Wu, Katrukha, & Akhmanova, 2018; Wu et al., 2016). Consistent with these findings, our microtubule regrowth assays in 2D MDCK cells (see Author response image 1) demonstrated that centriole depletion markedly altered microtubule organization. Control cells displayed the typical radial microtubule array emanating from a centralized centrosome, whereas centrinone-treated cells exhibited a dispersed microtubule network with enhanced microtubule growth from Golgi-associated sites.

**Author response image 1.** Staining of control or centrinone (CN) treated MDCK cells for α-tubulin (magenta), Golgi GM130 green and DNA (DAPI, blue) 1 min after nocodazole washout. Z-maximum projections of confocal images. The boxed cells in the overview images are magnified, and the microtubule regrowth regions are further enlarged. Scale bars: 50 μm (overview images), 10 μm (magnified cells), and 5 μm (enlarged microtubule regrowth regions).

(2) Disruption of centriole or centrosomal proteins

Previous studies have reported that depletion of the subdistal appendage protein ODF2 reduces centrosome–microtubule interactions and destabilizes centrosomal microtubules (Hung, Hehnly, & Doxsey, 2016; Ibi et al., 2011; Tateishi et al., 2013). In addition, in cells lacking the PCM protein pericentrin (PCNT), AKAP450 has been shown to partially compensate for centrosomal microtubule nucleation activity, although at a somewhat reduced level compared with wild-type cells (Figure 4—figure supplement 3A, B) (Gavilan et al., 2018).

(3) Inhibition of centrosomal microtubule nucleation

For dominant-negative Cdk5RAP2 and NEDD1 constructs, a previous study demonstrated that these constructs displace γ-tubulin from centrosomes and impair centrosomal microtubule nucleation (Vinopal et al., 2023). Consistent with this report, our MDCK cells expressing dominant-negative Cdk5RAP2 or NEDD1 also exhibited reduced γ-tubulin localization at centrioles (Figure 4—figure supplement 3C, D, and G).

Together, these findings support the interpretation that centrosomal microtubules are not strictly required for polarized vesicle trafficking, centrosome migration, or epithelial polarization, but instead enhance the efficiency and robustness of these processes. We agree with the reviewer that future studies using advanced imaging approaches under 3D culture conditions will be important to resolve the spatial organization and dynamics of the intracellular cytoskeleton during epithelial polarization.

**Reviewer #3 (Public review):**

Here the Wang et al resubmit their manuscript describing the events in the establishment of polarity in MDCK cells cultured in vitro. As with the original version, the description is throughout and is important to the field to report as it establishes a hierarchy of events in polarization, placing Par3 upstream of centrosome positioning and apical membrane component trafficking. Unfortunately, in the revised version, the authors addressed almost none of my points. They did a cursory job of responding in the rebuttal letter but made little attempt to actually address what was being asked or to incorporate any of my suggestions into the manuscript. The particularly egregious examples are cited below:

Comments on revisions:

(1) My original main experimental concern was not addressed: I had originally asked what role microtubules play in the process of polarization (either centrosomal or non-centrosomal). An obvious model is that Gp135, Rab11, etc. are delivered to the AMIS on centrosomal microtubules. Centrosomes might also be pulled to the AMIS via cortically derived microtubules as is the case in the *C. elegans* intestine where the centrosome moves apically on apical microtubules via dynein directed transport to the cortically anchored minus ends. The authors do not explore the role of microtubules in the revision, citing that it was not possible to observe the microtubules directly or to perform nocodazole experiments during polarization.

Instead, the authors use a relatively new genetic tool to disrupt centrosomal microtubules. They appear to succeed in displacing centrosomal g-tubulin using this tool, but without being able to observe microtubules, a remaining caveat of this experiment is that it is still unclear whether the authors have removed centrosomal microtubules. Compounding this issue is that this tool has never been used in MDCK cells. The authors conclude "we found that cells lacking centrosomal microtubules were still able to polarize and position the centrioles apically.", but they have not shown this, instead the data suggest this conclusion and the authors should acknowledge the caveat that they have no idea whether centrosomal microtubules are abolished.

We appreciate the reviewer’s important comments regarding the role of microtubules during epithelial polarization. We agree that determining how centrosomal and/or non-centrosomal microtubules contribute to apical trafficking and centrosome positioning represents an important mechanistic question.

We previously attempted to directly visualize microtubules during live imaging using SPY-tubulin labeling (see Author response image 2). However, under 3D Matrigel culture conditions, MDCK cells rapidly become rounded and densely packed, substantially reducing image contrast and making the majority of intracellular microtubule networks difficult to resolve, except for spindle microtubules and the cytokinetic bridge. In addition, nocodazole treatment caused mitotic arrest under our experimental conditions, thereby preventing de novo polarization from proceeding and precluding interpretation of polarity establishment.

**Author response image 2.** Time-lapse maximum-intensity z-projections of MDCK cells expressing EGFP-PACT (yellow; centrosome marker) and H2B-mCherry (magenta; nuclei) embedded in Matrigel. Microtubules were labeled with SiR-tubulin (cyan) before live-cell imaging. Images show a representative dividing cell. Time stamps indicate hours and minutes relative to anaphase onset (0:00). Scale bar, 10 μm.

To partially address the role of centrosomal microtubules, we used dominant-negative Cdk5RAP2 and NEDD1 constructs that have previously been shown to displace γ-tubulin from centrosomes and impair centrosomal microtubule nucleation (Vinopal et al., 2023). Consistent with this study, we observed substantial loss of γ-tubulin from centrioles in MDCK cells expressing these constructs (Figure 4— figure supplement 3C, D, and G). However, as the reviewer correctly points out, we were unable to directly visualize centrosomal microtubules under our 3D imaging conditions. Therefore, we cannot definitively conclude that centrosomal microtubules were completely abolished. We have revised the manuscript to clarify this limitation and to more cautiously state that our data suggest centrosomal microtubules may not be strictly required, for apical polarization and centrosome positioning under these conditions.

Similarly, the authors also state: "Additionally, although PCNT knockout cells show reduced microtubule nucleation ability, they still recruit a small amount of γ-tubulin". Where are the data that show that microtubule nucleation is reduced in these PCNT knock out cells?

We thank the reviewer for this comment and apologize for not sufficiently presenting these data in the previous revision. To directly assess microtubule nucleation activity in PCNT-KO cells, we performed microtubule regrowth assays in MDCK cells following nocodazole washout (Figure 4— figure supplement 3A, B). Compared with wild-type cells, PCNT-KO cells showed reduced centrosomal microtubule regrowth, indicating impaired microtubule nucleation capacity.

Importantly, microtubule nucleation was not completely abolished in PCNT-KO cells, consistent with previous reports showing that AKAP450 can partially compensate for the loss of pericentrin and maintain residual centrosomal microtubule nucleation activity (Gavilan et al., 2018).

(2) Many of my comments were addressed in the rebuttal, but not in the text.

We sincerely thank the reviewer for the valuable suggestions. We have carefully considered all comments and incorporated many of the recommended revisions into the revised manuscript. However, we found that including every additional analysis, experiment, and discussion in the main manuscript would substantially reduce its coherence and readability. Therefore, while not all new analyses and experimental results are included in the revised manuscript, we have addressed every comment comprehensively in this response letter. Where necessary, we performed additional experiments and analyses to obtain the requested data, and the corresponding results and explanations are provided in our responses. We hope the reviewer will understand our effort to thoroughly address all comments while preserving the clarity and overall flow of the manuscript.

The non-centrosomal GP135 in Figure 2 is not acknowledged or explained.

We apologize for not sufficiently addressing the non-centrosomal Gp135 signal in Figure 2. We have now revised the manuscript to explicitly describe and discuss this point in the text (Page 5, Paragraph 4).

That the polarity index does not actually measure polarity, but nuclear-centrosome distance is not acknowledged or explained in the paper.

We have revised the manuscript to explicitly state that the “polarity index” represents the distance between the nucleus and the centrosome, which we use as a quantitative indicator of the degree of cell polarity (Page 5, Paragraph 1).

I still don't believe that the quantification in Figure 3D matches the images I am being shown in Figure 3A. In the centrinone treatment condition, there is certainly an enrichment of GP135 at the AMIS that is not detected in the quantification. The method described in the rebuttal might miss this enrichment if it is offset from line drawn between the centroid of the two nuclei.

We thank the reviewer for this comment. To better address this concern, we have now included a 3D view of the corresponding image data (see Author response image 3 and Author response image 4). This analysis clarifies that, in the centrinone-treated condition, the Gp135 signal is not localized at the geometric center of the cell doublet, but is instead offset from the axis used in our line-scan quantification. As a result, the enrichment visible in the projection image was not fully captured by the original quantification method. SiR-DNA Gp135

**Author response image 3.** 

**Author response image 4.** 3D reconstructions of p53-KO control and centrinone-treated MDCK cell doublets expressing EGFP-Gp135. Images are shown after 90° rotations about the x-axis (or y-axis) to visualize the spatial distribution of Gp135. Fluorescence intensity profiles of EGFP-Gp135 were measured along the line connecting the two nuclei. White arrows indicate the central fluorescence intensity value used to quantify Gp135 accumulation at the apical membrane initiation site (AMIS) (a.u., arbitrary units). Time stamps indicate hours and minutes.

Cell height changes in the centrosome depleted cysts are still referenced in the text ("the cell heights of the centrosome-depleted cysts are less uniform"), but no specific data or image is called out. Currently, Figure 3G is referenced, but that is a graph of GP135 intensity

We have revised the manuscript to indicate representative images, ensuring that the text and figures are consistent (Page 7, Paragraph 3).

In my original review, I called on the authors to comment on the striking similarity of the mechanisms they documented in MDCK cells to what has been shown in in vivo systems. The authors did not do this, instead restating in the rebuttal some features of what they found. But, the mechanisms shown here are remarkably similar to the polarization of primordia that generate tubular organs in vivo. Perhaps most striking is the similarity to the *C. elegans* intestine where Par3 localizes to the cortex at the site of an apical MTOC that pulls the centrosome to the apical surface via dynein (Feldman and Priess, 2012). Instead of discussing this similarity, the authors state: "Par3 is likely to regulate centrosome positioning through some intermediate molecules or mechanisms, but its specific mechanism is still unclear and requires further investigation." Given the acetylated tubulin signal emanating from the Par3 positive patch in Figure 5E and F, I suspect similar mechanisms to the *C. elegans* intestine are at play here. Such a parallel should be noted in the Discussion.

We thank the reviewer for this insightful suggestion. In the revised manuscript, we have expanded the Discussion section to compare our findings with epithelial polarization mechanisms described in in vivo systems, including the *C. elegans* intestine and other tubular epithelial tissues (Page 13, Paragraph 2).

We agree that the hierarchical relationship we observe between Par3 localization, centrosome positioning, and apical membrane formation bears important conceptual similarities to mechanisms reported in the *C. elegans* intestine (Feldman & Priess, 2012). We also considered the possibility that Par3 may regulate centrosome positioning through dynein-dependent mechanisms.

To examine this possibility, we performed immunofluorescence staining for the dynein cofactor dynactin subunit p150^Glued^. However, we did not observe enrichment of p150^Glued^ at the center of cell doublets during the cytokinetic pre-abscission stage (see Author response image 5), suggesting that dynein is not strongly concentrated together with Par3 near the AMIS under our conditions.

In addition, pharmacological inhibition of dynein resulted in cytokinesis failure and the formation of binucleated cells, preventing reliable assessment of centrosome migration and polarity establishment.

We would also like to clarify that the acetylated tubulin signal observed in Figure 5E and F does not emanate from the Par3-positive patch. Rather, this signal corresponds to the cytokinetic bridge, adjacent to which Par3 accumulates during cytokinesis. Consistent with this interpretation, γ-tubulin was not detected at the Par3-positive region (Figure 1A).

**Author response image 5.** Single MDCK cells after 12 h of culture in Matrigel. Immunostaining signals of the indicated markers are shown: centrosome marker PACT-mKO1, dynactin subunit p150Glued, Gp135, and DAPI. Single confocal sections through the middle of a cyst are shown. The order of polarization is arranged from single cell (1-cell), metaphase (Meta), telophase (Telo), cytokinetic pre-abscission (Pre-Abs), post-cytokinesis (Post-CK), to lumen open (LO). Scale bar: 5 μm.

I had originally commented that "I find the results in Figure 6G puzzling. Why is ECM signaling required for Gp135 recruitment to the centrosome. Could the authors discuss what this means?" The authors responded that "The data in Figure 6G do not indicate that ECM signaling is required for the recruitment of Gp135 to the centrosome". In Figure 6G, the localization of GP135 to the centrosome appears significantly delayed compared to its localization to the centrosome in images where cells were cultured in Matrigel.

Indeed, the authors argue that the centrosomal localization precedes and contributes to its localization to the AMIS. In the absence of ECM, GP135 localizes to the membrane before it localizes to the centrosome and its localization to the centrosome appears significantly reduced. Thus, my original and current interpretation is that ECM signaling is somehow required for the centrosomal targeting of GP135. One could make a competition argument, i.e. that the cortex in the absence of ECM is somehow a more desirable place to localize than the centrosome, but this experiment also argues that the centrosome does not need to be a source of this material in order for it to end up on the cortex.

We agree that the absence of ECM substantially alters the trafficking behavior of Gp135.

Our interpretation is that ECM primarily promotes the endocytosis and internal trafficking of Gp135, thereby enabling its redistribution to membrane domains lacking ECM contact and facilitating AMIS formation (Buckley & St Johnston, 2022; O'Brien et al., 2001; Yu et al., 2005).

Under ECM-free conditions, a larger fraction of Gp135 remains associated with the plasma membrane, resulting in reduced internalized Gp135 available for centrosome-associated trafficking. During anaphase to telophase (Figure 6G, 0:05–0:20), Gp135 predominantly redistributes along the plasma membrane toward the cleavage furrow. Only after cytokinesis initiation (Figure 6G, 0:30) do we observe a small amount of internalized Gp135 associated with centrosomes near the center of the cell doublet.

Importantly, we agree with the reviewer that these findings suggest centrosomal trafficking is not absolutely required for Gp135 to localize to the plasma membrane. Rather, our data support a model in which centrosome-associated trafficking contributes specifically to the efficient and spatially restricted delivery of Gp135 to the AMIS during epithelial polarization.

We have revised the manuscript to clarify this interpretation and to avoid overstating the role of centrosome-associated Gp135 trafficking.

(3) There needs to be precision in the language used in many places:

I don't understand this line in the abstract: "When cultured in Matrigel, de novo polarization of a single epithelial cell is often coupled with mitosis." If a cell has divided, it is no longer a single cell.

We have revised the sentence to: “When cultured in Matrigel, de novo polarization of a single epithelial cell is often coupled with cytokinesis (Page 1, Paragraph 1).” This indicates that polarization happens as the cell divides. We thank the reviewer for this helpful suggestion, which has improved the readability of the sentence.

The authors state in the Introduction "Because of its strong ability to nucleate microtubules, the centrosome functions as the primary microtubule organizing center", but then state ""In polarized epithelial cells, the centrosome is localized at the apical region during interphase, which contributes to the construction of an asymmetric microtubule network conducive to polarized vesicle trafficking". In the latter statement, I assume the authors are describing the well-characterized apical microtubule network in epithelial cells that is non-centrosomal. Thus, the latter sentence is at odds with the former.

We did not intend to refer to the apical non-centrosomal microtubule network present in mature, fully polarized epithelial cells. Rather, we were referring to the off-center centrosome functions as an off-center MTOC, creating an asymmetric microtubule network during the early stages of epithelial polarization, as mentioned in previous review papers (Meiring, Shneyer, & Akhmanova, 2020).

The apical non-centrosomal microtubule network is a feature of mature, fully polarized epithelial cells. In fact, its formation is also driven by the release of microtubule minus-ends from the off-centre centrosome, which are then transferred to the apical membrane (Goldspink et al., 2017; Moss et al., 2007; Sanchez & Feldman, 2017).

The authors continually refer to Par3 as a tight junction protein. "Par3, which controls tight junction assembly to partition the apical surface from the basolateral surface". To my knowledge, PARD3 is an apical protein with similar localization to *C. elegans* PAR-3 and Drosophila Bazooka. PARD3B is a junctional protein. I assume that the antibody that the authors are using is to PARD3 and not PARD3B? Can the authors please clarify this in the text?

The antibody used for PARD3 staining was the Merck Millipore rabbit polyclonal antibody (Cat. No. 07-330), generated against a GST-tagged recombinant fragment corresponding to 288 amino acids from the internal region of mouse PAR-3.

Canine PARD3 and PARD3B are encoded by distinct genes located on chromosomes 2 and 37, respectively. In our study, we used two independent shRNA constructs specifically targeting canine PARD3, both of which reduced the immunoblot signal detected by this antibody, supporting the conclusion that the antibody primarily recognizes PARD3 rather than PARD3B.

However, in immunofluorescence staining, the signal was mainly localized at tight junctions, and we did not observe significant signal at the apical membrane. We will further clarify in the revised manuscript that this antibody targets PARD3 rather than PARD3B (Page 10, Paragraph 1).

Reference

Buckley, C. E., & St Johnston, D. (2022). Apical-basal polarity and the control of epithelial form and function. Nat Rev Mol Cell Biol, 23(8), 559–577. doi:10.1038/s41580-022-00465-y

Chen, F., Wu, J., Iwanski, M. K., Jurriens, D., Sandron, A., Pasolli, M., . . . Akhmanova, A. (2022). Self-assembly of pericentriolar material in interphase cells lacking centrioles. Elife, 11. doi:10.7554/eLife.77892

Feldman, J. L., & Priess, J. R. (2012). A role for the centrosome and PAR-3 in the hand-off of MTOC function during epithelial polarization. Curr Biol, 22(7), 575–582. doi:10.1016/j.cub.2012.02.044

Gavilan, M. P., Gandolfo, P., Balestra, F. R., Arias, F., Bornens, M., & Rios, R. M. (2018). The dual role of the centrosome in organizing the microtubule network in interphase. EMBO Rep, 19(11). doi:10.15252/embr.201845942

Goldspink, D. A., Rookyard, C., Tyrrell, B. J., Gadsby, J., Perkins, J., Lund, E. K., . . . Mogensen, M. M. (2017). Ninein is essential for apico-basal microtubule formation and CLIP-170 facilitates its redeployment to noncentrosomal microtubule organizing centres. Open Biol, 7(2). doi:10.1098/rsob.160274

Hung, H. F., Hehnly, H., & Doxsey, S. (2016). The Mother Centriole Appendage Protein Cenexin Modulates Lumen Formation through Spindle Orientation. Curr Biol, 26(6), 793–801. doi:10.1016/j.cub.2016.01.025

Ibi, M., Zou, P., Inoko, A., Shiromizu, T., Matsuyama, M., Hayashi, Y., . . . Inagaki, M. (2011). Trichoplein controls microtubule anchoring at the centrosome by binding to Odf2 and ninein. J Cell Sci, 124(Pt 6), 857–864. doi:10.1242/jcs.075705

Martin, M., Veloso, A., Wu, J., Katrukha, E. A., & Akhmanova, A. (2018). Control of endothelial cell polarity and sprouting angiogenesis by non-centrosomal microtubules. Elife, 7. doi:10.7554/eLife.33864

Meiring, J. C. M., Shneyer, B. I., & Akhmanova, A. (2020). Generation and regulation of microtubule network asymmetry to drive cell polarity. Curr Opin Cell Biol, 62, 86–95. doi:10.1016/j.ceb.2019.10.004

Moss, D. K., Bellett, G., Carter, J. M., Liovic, M., Keynton, J., Prescott, A. R., . . . Mogensen, M. M. (2007). Ninein is released from the centrosome and moves bi-directionally along microtubules. J Cell Sci, 120(Pt 17), 3064–3074. doi:10.1242/jcs.010322

O'Brien, L. E., Jou, T. S., Pollack, A. L., Zhang, Q., Hansen, S. H., Yurchenco, P., & Mostov, K. E. (2001). Rac1 orientates epithelial apical polarity through effects on basolateral laminin assembly. Nat Cell Biol, 3(9), 831–838. doi:10.1038/ncb0901-831

Sanchez, A. D., & Feldman, J. L. (2017). Microtubule-organizing centers: from the centrosome to noncentrosomal sites. Curr Opin Cell Biol, 44, 93–101. doi:10.1016/j.ceb.2016.09.003

Tateishi, K., Yamazaki, Y., Nishida, T., Watanabe, S., Kunimoto, K., Ishikawa, H., & Tsukita, S. (2013). Two appendages homologous between basal bodies and centrioles are formed using distinct Odf2 domains. J Cell Biol, 203(3), 417–425. doi:10.1083/jcb.201303071

Vinopal, S., Dupraz, S., Alfadil, E., Pietralla, T., Bendre, S., Stiess, M., . . . Bradke, F. (2023). Centrosomal microtubule nucleation regulates radial migration of projection neurons independently of polarization in the developing brain. Neuron, 111(8), 1241–1263 e1216. doi:10.1016/j.neuron.2023.01.020

Wu, J., de Heus, C., Liu, Q., Bouchet, B. P., Noordstra, I., Jiang, K., . . . Akhmanova, A. (2016). Molecular Pathway of Microtubule Organization at the Golgi Apparatus. Dev Cell, 39(1), 44–60. doi:10.1016/j.devcel.2016.08.009

Yu, W., Datta, A., Leroy, P., O'Brien, L. E., Mak, G., Jou, T. S., . . . Zegers, M. M. (2005). Beta1-integrin orients epithelial polarity via Rac1 and laminin. Mol Biol Cell, 16(2), 433–445. doi:10.1091/mbc.e04-05-0435
